# Supplementary material for: Epigenomic perturbation of novel EGFR enhancers reduces the proliferative and invasive capacity of glioblastoma and increases sensitivity to temozolomide
Source: BMC Cancer. 2023 Oct 6;23:945. doi: 10.1186/s12885-023-11418-9 (PMC10557167; doi:10.1186/s12885-023-11418-9)
Supplement: Supplementary file 1 — Additional file 1: Supplementary Figure 1. Recruitment of dCas9-KRAB repressor complex leads to enrichment of H3K9me3 at specic targeted sites in U251 cells. A-F, Bar charts depicting the enrichment of H3K9me3, as determined by ChIP-qPCR, at each genomic region (i.e., CE5B, CE6B, CE8, Promoter) and in each of the EGFR enhancer-repressed lines: iCE5B (B), iCE6B (C), iCE5B+6B (D) and iCE8 (E), alongside the iPromoter (F) and control line (A). Supplementary Figure 2. CRISPRi of EGFR enhancer CE5B+6B in Human Glioblastoma cell line U3013 downregulates EGFR gene expression, reduces cell proliferation rate, and sensitises cells to temozolomide (TMZ). A, EGFR gene expression as determined by RT-qPCR. B, Proliferation rates of the iCE5B+6B and promoter-repressed U3013 cell lines determined by live-cell imaging. Images were acquired every 4 hours and proliferation was determined by automatic cell count. Data is normalised to t=0 and presented as mean ± SEM (n=3). Statistical significance was assessed by unpaired t test (* P < 0.05, ** P < 0.01). C-D, Proliferation rates of control U3013 and iCE5B+6B enhancer-repressed U3013 cells determined by live-cell imaging upon treatment with 125μM TMZ in comparison with the DMSO-treated control. Images were acquired every 4 hours and proliferation was determined by automatic cell count. Data is normalised to t=0h and represented as mean ± SEM (n=4). P values were determined by unpaired t test. Supplementary Figure 3. CRISPR/Cas9-mediated deletion of EGFR enhancers in U251 cells downregulates EGFR gene expression and affects cell proliferation rates. A, Genotyping PCR of the EGFR enhancer-deleted cell lines (ΔCE5B, ΔCE6B, ΔCE5B+6B, ΔCE8) alongside the Δ Promoter and empty vector control lines (left), and schematic outline of the PCR genotyping strategy (right). Note that the wild-type CE5B+6B allele is too large to be amplied under these conditions. Full uncropped gel image presented in Supplementary Figure 6. B, EGFR gene expression le [file 12885_2023_11418_MOESM1_ESM.pdf]

Epigenomic perturbation of novel *EGFR* enhancers reduces the proliferative and invasive capacity of glioblastoma and increases sensitivity to temozolomide

Craig A. Vincent[1,2], Itzel Nissen[1,2], Soran Dakhel[1,2], Andreas Hörnblad[1] and Silvia Remeseiro[1,2,#]

[1] Umeå Centre for Molecular Medicine (UCMM), Umeå University, Umeå, Sweden.

[2] Wallenberg Centre for Molecular Medicine (WCMM), Umeå University, Umeå, Sweden.

# Corresponding author (lead contact): silvia.remeseiro@umu.se

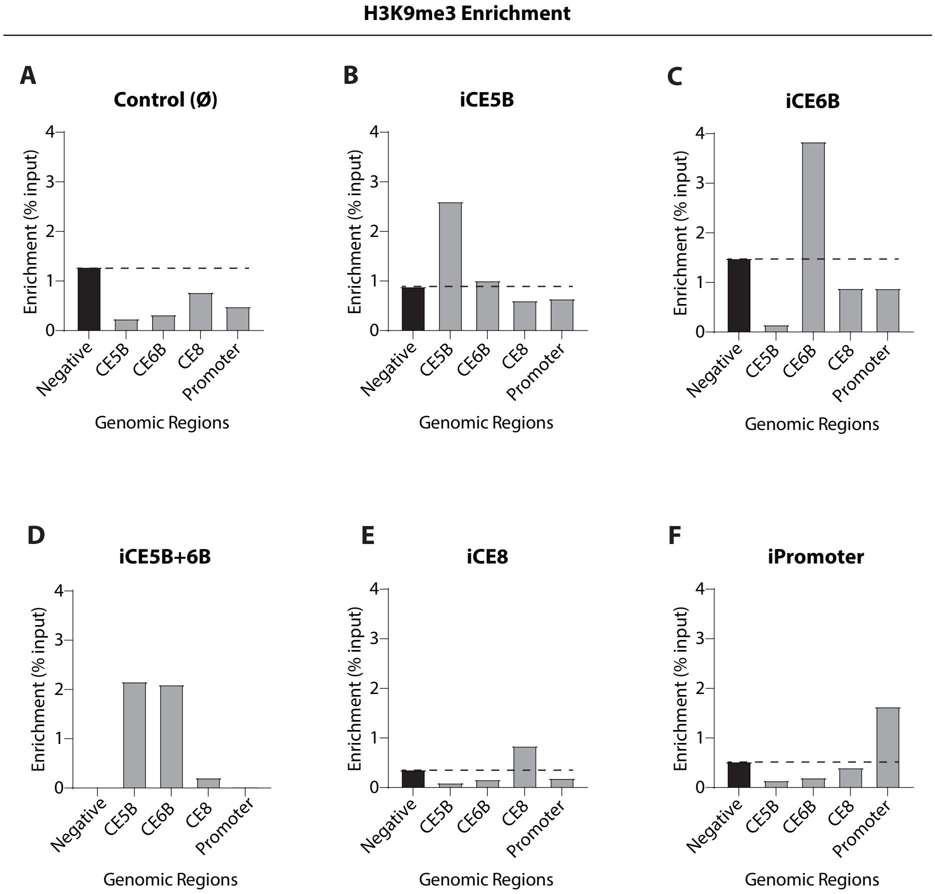

Supplementary Figure 1. Recruitment of dCas9-KRAB repressor complex leads to enrichment of H3K9me3 at specific targeted sites in U251 cells. **A-F,** Bar charts depicting the enrichment of H3K9me3, as determined by ChIP-qPCR, at each genomic region (i.e. CE5B, CE6B, CE8, Promoter) and in each of the *EGFR* enhancer-repressed lines: iCE5B (**B**), iCE6B (**C**), iCE5B+6B (**D**) and iCE8 (**E**), alongside the iPromoter (**F**) and control line (**A**).

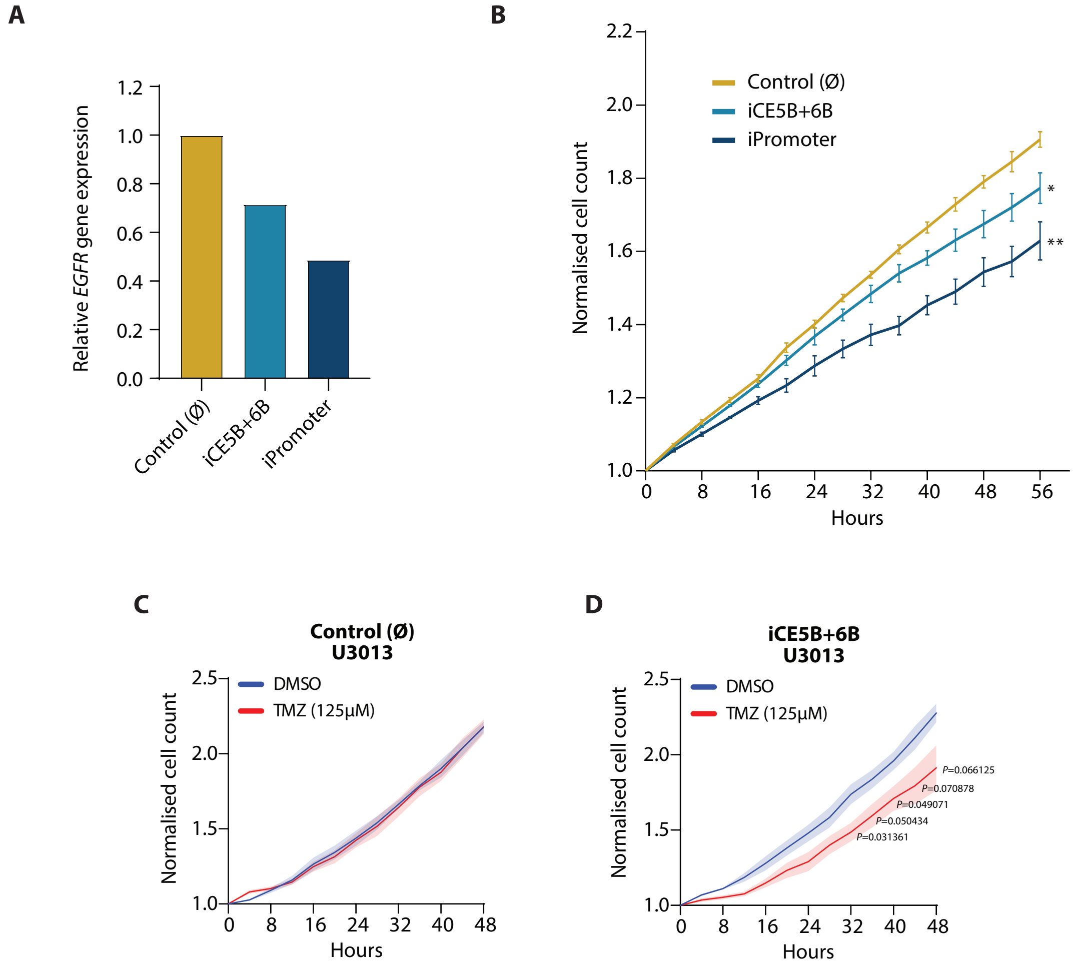

Supplementary Figure 2. CRISPRi of *EGFR* enhancer CE5B+6B in Human Glioblastoma cell line U3013 downregulates *EGFR* gene expression, reduces cell proliferation rate, and sensitises cells to temozolomide (TMZ). **A**, *EGFR* gene expression as determined by RT-qPCR. **B**, Proliferation rates of the iCE5B+6B and promoter-repressed U3013 cell lines determined by live-cell imaging. Images were acquired every 4 hours and proliferation was determined by automatic cell count. Data is normalised to t=0 and presented as mean ± SEM (n=3). Statistical significance was assessed by unpaired *t* test (* $P < 0.05$, ** $P < 0.01$). **C-D,** Proliferation rates of control U3013 and iCE5B+6B enhancer-repressed U3013 cells determined by live-cell imaging upon treatment with 125μM TMZ in comparison with the DMSO-treated control. Images were acquired every 4 hours and proliferation was determined by automatic cell count. Data is normalised to t=0h and represented as mean ± SEM (n=4). *P* values were determined by unpaired *t* test.

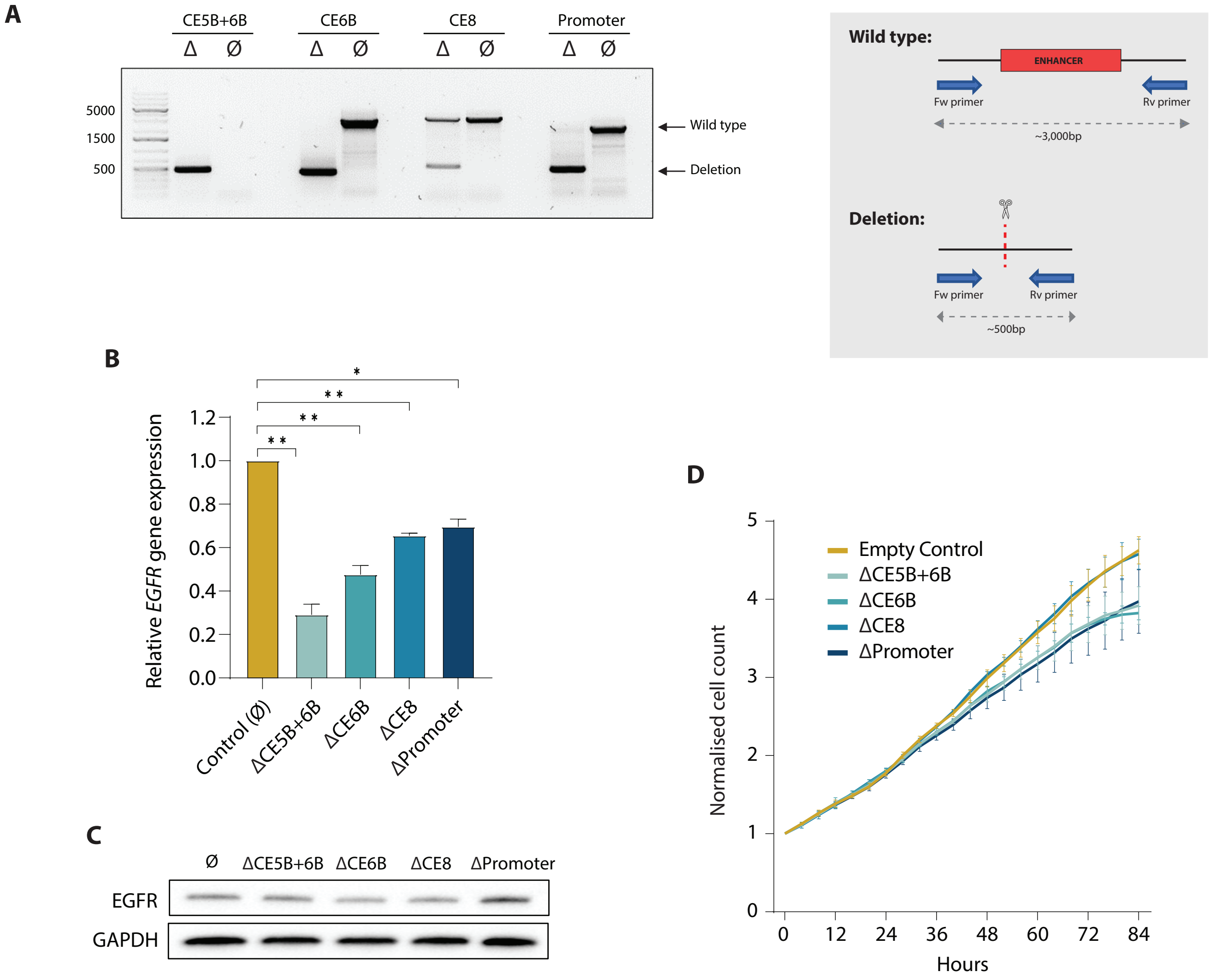

Supplementary Figure 3. CRISPR/Cas9-mediated deletion of *EGFR* enhancers in U251 cells downregulates *EGFR* gene expression and affects cell proliferation rates. **A**, Genotyping PCR of the *EGFR* enhancer-deleted cell lines (ΔCE5B, ΔCE6B, ΔCE5B+6B, ΔCE8) alongside the Δ Promoter and empty vector control lines (left), and schematic outline of the PCR genotyping strategy (right). Note that the wild-type CE5B+6B allele is too large to be amplified under these conditions. Full uncropped gel image presented in Supplementary Figure 6. **B**, *EGFR* gene expression levels relative to *HPRT* in *EGFR* enhancer-deleted cell lines as determined by RT-qPCR assays. Data is represented as mean ± SEM (n=3). Statistical significance as assessed by unpaired *t* test with Welch's correction (* $P < 0.05$, ** $P < 0.01$). **C**, Cropped western blots showing EGFR protein expression and normalised to GAPDH protein levels (full uncropped blots in Supplementary Figure 6). **D**, Proliferation rates of cell lines carrying *EGFR* enhancer deletions or promoter deletions as determined by live-cell imaging, and in comparison to the empty vector control line. Images were acquired every 4 hours and proliferation was determined by automatic cell count. Data is normalised to t= 0h and plotted as mean ± SEM (n=3).

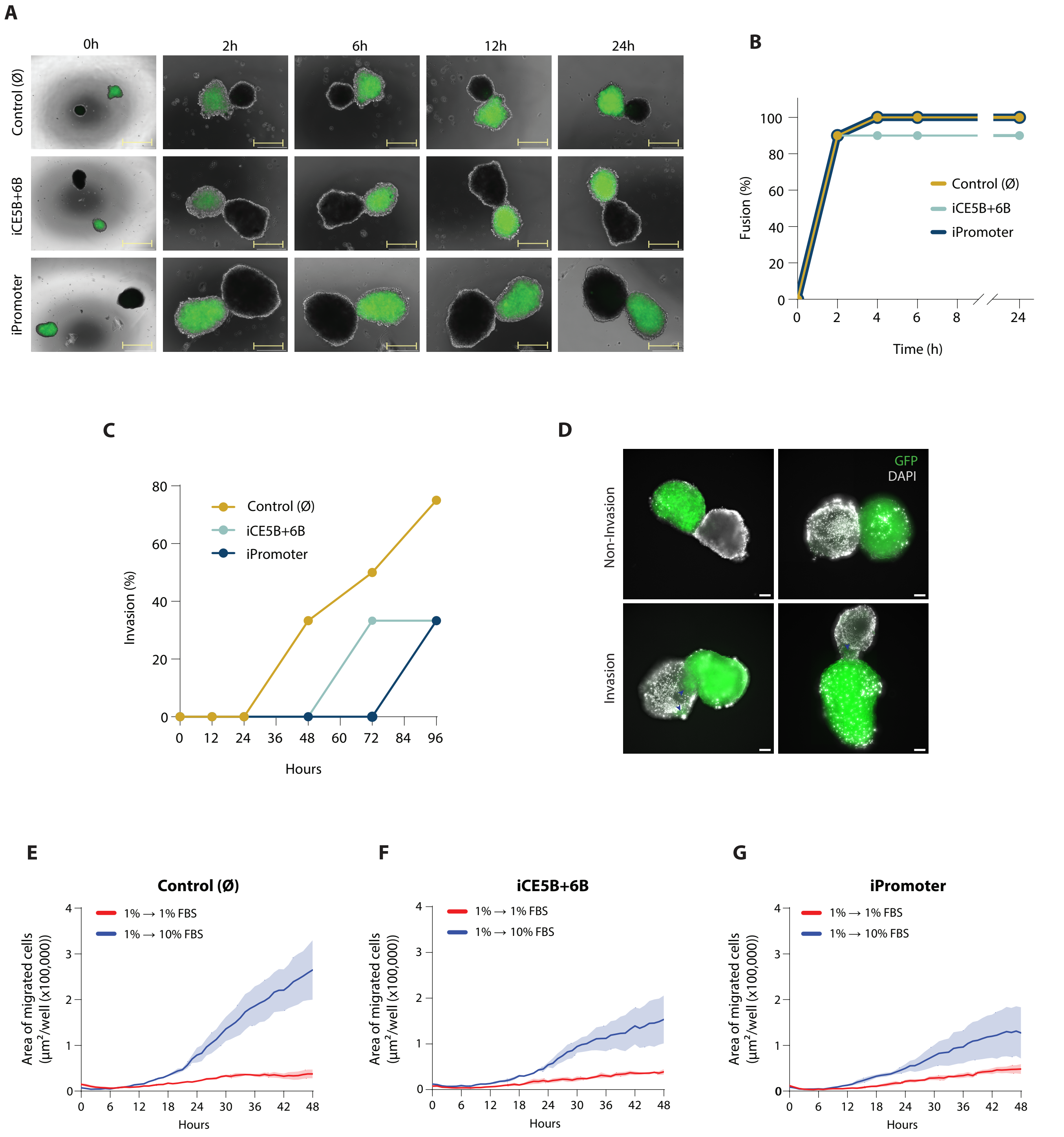

Supplementary Figure 4. CRISPRi of the *EGFR* enhancer CE5B+6B and promoter compromises the invasive capacity of U251 cells. **A,** Representative images of the fusion between the fetal brain spheroids and the GFP+ tumour spheres generated from control, iCE5B+6B and iPromoter lines. Scale bars 750µm (0h) and 300µm (2-24h). **B,** Quantification of fusion events across a pool of independent tumour sphere/brain spheroid confrontation assays (n=10). **C,** Quantification of invasion frequency between the GFP+ tumour spheres and brain spheroids between 0h and 96h. **D,** Representative images of invasion/non-invasion between GFP+ tumour spheres and brain spheroids. Scale bar 100µm. Arrowheads point to areas of invasion. **E-F**, Line plots comparing the rate at which the respective cell lines migrate either from media containing 1% FBS to 1% FBS (no-chemoattractant negative control) or from 1% FBS to 10% FBS (chemoattractant condition). Migration was assessed by live-cell imaging taking images every hour and migration rate was determined by automatic quantification of the area of migrated cells. Data is represented as mean ± SEM (n=3).

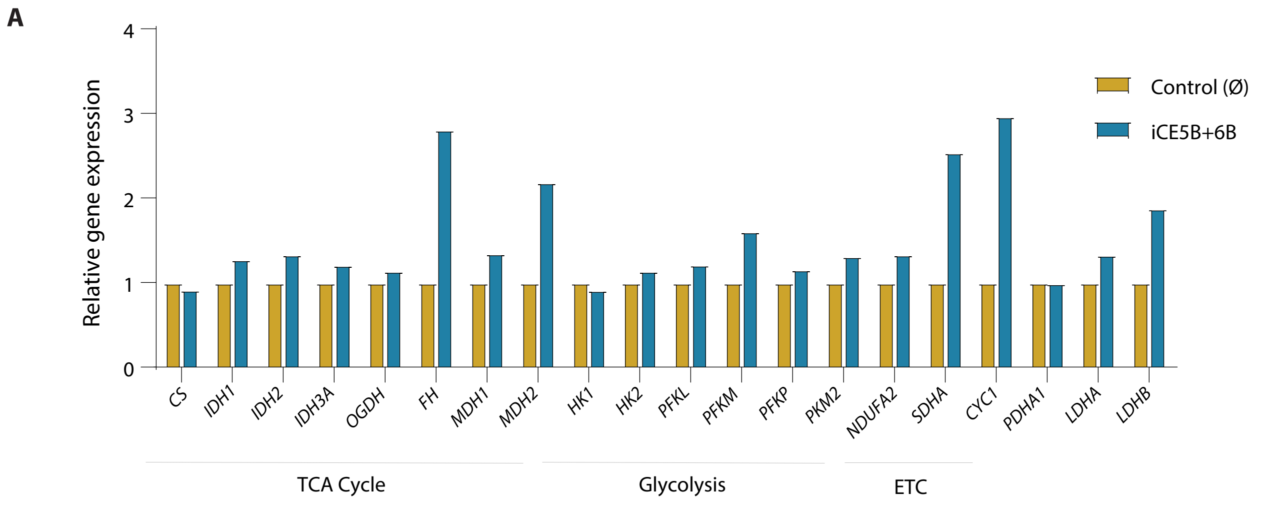

Supplementary Figure 5. Expression of key metabolic genes in the *EGFR* enhancer-repressed iCE5B+6B cell line. **A,** Results from qPCR analysis where certain metabolic genes are upregulated in the iCE5B+6B line relative to the control. Gene expression levels are relative to *HPRT.*

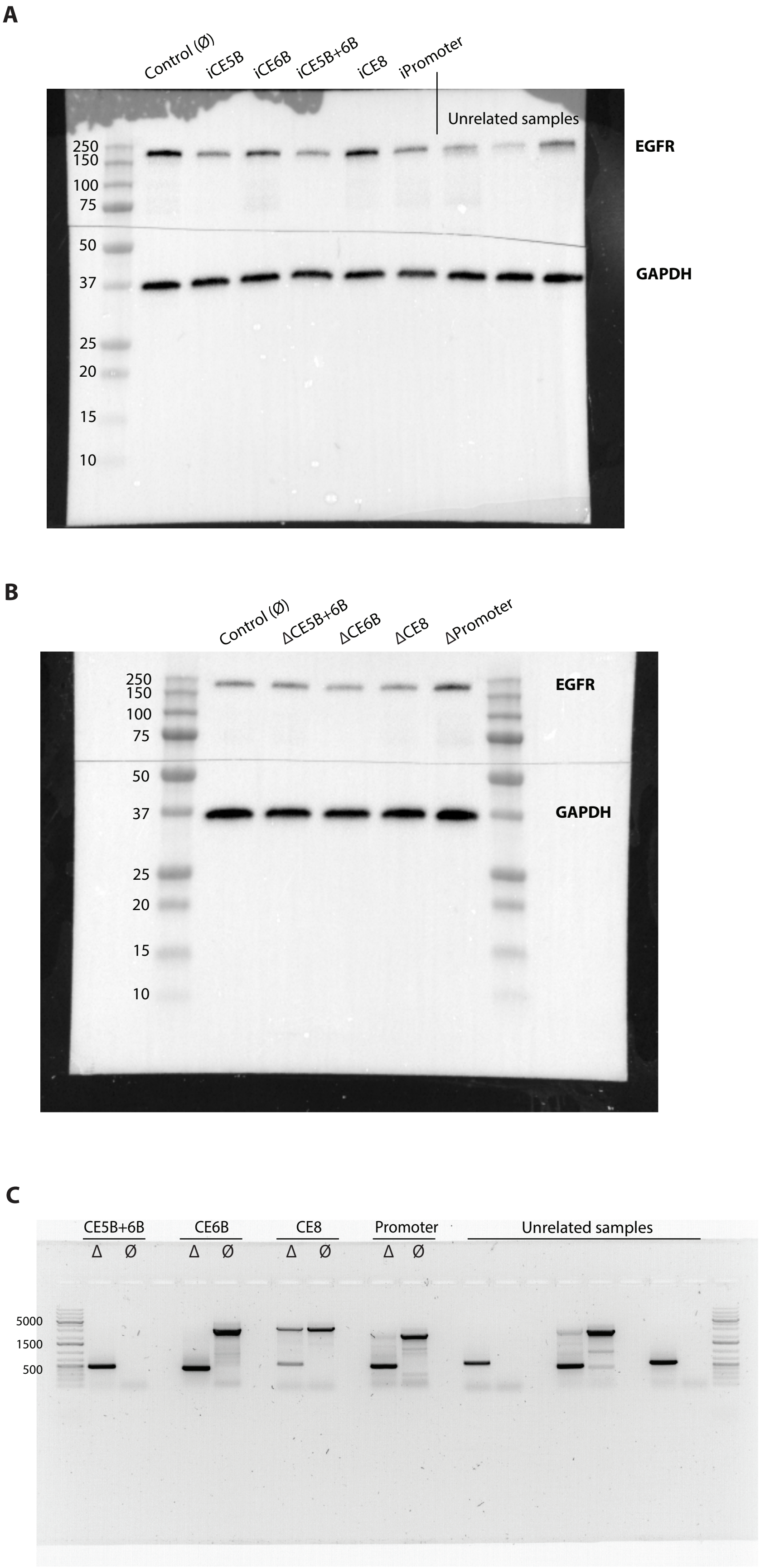

Supplementary Figure 6. Uncropped western blot and gel images. **A**, Western blot on repressed cell lines (**A**) and deletion cell lines (**B**) detecting EGFR and GAPDH expression. Cropped images presented in Figure 1G and Supplementary Figure 3C, respectively. **C,** Uncropped genotyping gel from enhancer-deletion U251 cell lines. Cropped image presented in Supplementary Figure 3A.

Supplementary Table S1

Purpose	Primer Name	Primer Sequence
Luciferase Dual-Reporter Assay	CE1 EGFR Fw	NNGGTACCAGCAGCCAGGACCATCTTTT
	CE1 EGFR Rv	NNAGATCTCAGGGAATGGGGAGGCTTTT
	CE2 EGFR Fw	NNGGTACCACCACAGAGCAGACCAACAG
	CE2 EGFR Rv	NNAGATCTAGGTCACTGAACCCTCCCTT
	CE3 EGFR Fw	NNGGTACCTCCTTGCCTGAAACCTGCAA
	CE3 EGFR Rv	NNAGATCTTCTTGGCCGTCCTTCATCAC
	CE4A EGFR Fw	NNGGTACCCCAGTCCAAGGTTAAAGGAAACTT
	CE4A EGFR Rv	NNAGATCTGAAGCCTCGGATTCACCAGC
	CE4B EGFR Fw	NNGGTACCTCTCGGAAAATAGCACCCTTCA
	CE4B EGFR Rv	NNAGATCTTGGATGAAGTCAGGGAAACCC
	CE4C EGFR Fw	NNGGTACCACCGAACATGTGCGCATTC
	CE4C EGFR Rv	NNAGATCTCTGGCGTTTTTCATTCCGTC
	CE5A EGFR Fw	NNGGTACCAAACGGACTTGTGGCATCTTT
	CE5A EGFR Rv	NNAGATCTCATTAAAGGCCCAGAATGCAGC
	CE5B EGFR Fw	NNGGTACCGTTCTTCCCCACTAGAAGCCAA
	CE5B EGFR Rv	NNAGATCTATGCCTCTGTGATGTGCGA
	CE5C EGFR Fw	NNGGTACCAGGTGTCTGACTGAGGCGTT
	CE5C EGFR Rv	NNAGATCTTAGAAGGATGGTGAGGATTGAGGA
	CE6A EGFR Fw	NNGGTACCCAGCAAACCTCCACTGCCTA
	CE6A EGFR Rv	NNAGATCTGTGCCCACCAGAAAATGCAG
	CE6B EGFR Fw	NNGGTACCCCACTTACCAGCTGTGGGAC
	CE6B EGFR Rv	NNAGATCTACTTCGGTGGCCTTTCACAT
	CE6C EGFR Fw	NNGGTACCACCAAGCACGGTGTTCTCTT
	CE6C EGFR Rv	NNAGATCTATGTCCAAGCAGAGGATGGC
	CE7A EGFR Fw	NNGGTACCTACCTTCTGTCTGCTGGCAC
	CE7A EGFR Rv	NNAGATCTGAAGAGGAGAGGACGAGGGA
	CE7B EGFR Fw	NNGGTACCCAGCTGAGGCCTACAGGAAC
	CE7B EGFR Rv	NNAGATCTAAATCCCGTGTGGTGGTCTC
	EGFR CE8 Fw	NNNNGGTACCAGGTGTCCAGTGTTGTCTGTG
	EGFR CE8 Rv	NNNNAGATCTGCTGGAAGGAAGTGCTGAGA
	EGFR CE9a Fw	NNNNGGTACCAGAATGAGCAGCACAGTCCC
	EGFR CE9a Rv	NNNNAGATCTCCGGATCCGAACAGGAAACA
	EGFR CE9b Fw	NNNNGGTACCGGTGTGAAGTCGCTGGAGAA
	EGFR CE9b Rv	NNNNAGATCTCTGCTGTGTGCTCATGGTTG
	EGFR CE10a Fw	NNNNGGTACCGAGGCCTTTGCAGAGGATGT
	EGFR CE10a Rv	NNNNAGATCTGACAGCTGTTAGCCTGGGAG
	EGFR CE10b Fw	NNNNGGTACCACACTTGGCACTTGTAGGCA
	EGFR CE10b Rv	NNNNAGATCTGGAGCATGACACTGAGGCTT
	EGFR Promoter Fw	NNNNGGTACCCTCCTCCCCTTTCACAGAGC
	EGFR Promoter Rv	NNNNAGATCTAAATGAGGGCACCCAACTCC

Supplementary Table S2

Purpose	Primer Name	Primer Sequence
ChIP-qPCR	ChIP ctrl #1 Fw	TGGACCAGACCGTAGAACCT
	ChIP ctrl #1 Rv	CATGGCCTGAGCAACAGGTA
NB:	ChIP ctrl #2 Fw	AACTCACCTACCCAACCGAC
ChIP ctrl #1:	ChIP ctrl #2 Rv	ATAGGACGGAGGAGTGGGC
- H3K27ac neg.	EGFR_promoter Fw_hu_ChIP	TATTGATCGGGAGAGCCGGA
- H3K27me3 pos.	EGFR_promoter Rv_hu_ChIP	TTCCTCCAGAGCCCGACT
- H3K9me3 pos.	EGFR_CE1 Fw_hu_ChIP	CCACCCCTTGCCTACTCATT
ChIP ctrl #2:	EGFR_CE1 Rv_hu_ChIP	GAAGAGAGACAGGCCACACC
- H3K27ac pos.	EGFR_CE3B Fw_hu_ChIP	GACAGGCAGTGGCTACACAT
- H3K27me3 neg.	EGFR_CE3B Rv_hu_ChIP	GCGTGCTGATGGGTGTTTTT
- H3K9me3 neg.	EGFR_CE4A Fw_hu_ChIP	AGGCTTTTGCTCACAGTGGT
	EGFR_CE4A Rv_hu_ChIP	CAGAACGGCTCCTTCACCTT
	EGFR_CE5A Fw_hu_ChIP	TACCATCAGCACACGCAGTT
	EGFR_CE5A Rv_hu_ChIP	ATGCCCATGACGTCCTTTGT
	EGFR_CE5C Fw_hu_ChIP	TGCAGAGGAGGTGTCTGACT
	EGFR_CE5C Rv_hu_ChIP	CCTGCTGACAGGGAAAGAGG
	EGFR_CE6B Fw_hu_ChIP	CACCCTTCCTGCTCACTCTG
	EGFR_CE6B Rv_hu_ChIP	TTTCCTCCTGGACCTGGACA
	EGFR_CE7B Fw_hu_ChIP	AGTGCCCATTTCTCTCCCAC
	EGFR_CE7B Rv_hu_ChIP	CTGCTTCTCACACTCCTGGG
	EGFR_CE8 Fw_hu_ChIP	GAATTCGGGAGCTGGTTGGA
	EGFR_CE8 Rv_hu_ChIP	ACGCCTCTCTGACAATGGTG
	EGFR_CE9 Fw_hu_ChIP	TCCTTTGGGCCTAGGATTGC
	EGFR_CE9 Rv_hu_ChIP	CCCAGAGCTCCCTCTTGTTC
	EGFR_CE10 Fw_hu_ChIP	ACAACATGTGAGCAGGAGGG
	EGFR_CE10 Rv_hu_ChIP	GGAGAGTCCCTGGTCAAAGC

Supplementary Table S3

Purpose	Primer Name	Primer Sequence
CRISPR gRNAs: Deletions	EGFR CE5B CRISPR1 Fw	CACCGAGGTTGTATGTAGTATCCAC
	EGFR CE5B CRISPR1 Rv	AAACGTGGATACTACATACAACCTC
	EGFR CE6B CRISPR1 Fw	CACCGCATTTCGTATGTGACCTGCA
	EGFR CE6B CRISPR1 Rv	AAACTGCAGGTCACATACGAAATGC
	EGFR CE8 CRISPR1 Fw	CACCGCTTCAAAGAACAAGTTACTC
	EGFR CE8 CRISPR1 Rv	AAACGAGTAACTTGTTCTTTGAAGC
	EGFR Promoter CRISPR1 Fw	CACCGAAGCGTTGCTGGACAAGAG
	EGFR Promoter CRISPR1 Rv	AAACCTCTTGTCCAGCAACGCTTC
	EGFR CE5B CRISPR2 Fw	CACCGTGGATTCACAAGTAAGCAAG
	EGFR CE5B CRISPR2 Rv	AAACCTTGCTTACTTGTGAATCCAC
	EGFR CE6B CRISPR2 Fw	CACCGTCATTCTAATTACCAAGCA
	EGFR CE6B CRISPR2 Rv	AAACTGCTTGGTAATTAGAATGAC
	EGFR CE8 CRISPR2 Fw	CACCGTACCGTGAGGATGTGGAGCG
	EGFR CE8 CRISPR2 Rv	AAACCGCTCCACATCCTCACGGTAC
	EGFR Promoter CRISPR2 Fw	CACCGCGGACTTTAGAGCACCACCT
	EGFR Promoter CRISPR2 Rv	AAACAGGTGGTGCTCTAAAGTCCGC
CRISPR gRNAs: KRAB repression	EGFR CE5B CRISPR KRAB 3 Fw	CACCGCTTCTTAACAATACAAGGA
	EGFR CE5B CRISPR KRAB 3 Rv	AAACTCCTTGTATTGTTAAGAAGC
	EGFR CE5B CRISPR KRAB 4 Fw	CACCGATACCGTGGTCATAATAGTG
	EGFR CE5B CRISPR KRAB 4 Rv	AAACCACTATTATGACCACGGTATC
	EGFR CE6B CRISPR KRAB 3 Fw	CACCGCCTTAAAAAGATAGTGCAGA
	EGFR CE6B CRISPR KRAB 3 Rv	AAACTCTGCACTATCTTTTTAAGGC
	EGFR CE6B CRISPR KRAB 4 Fw	CACCGACCCTTCCCCTAGTCTGGAG
	EGFR CE6B CRISPR KRAB 4 Rv	AAACCTCCAGACTAGGGGAAGGGTC
	EGFR CE8 CRISPR KRAB 3 Fw	CACCGGTGCAGAAGAGACACCGAG
	EGFR CE8 CRISPR KRAB 3 Rv	AAACCTCGGTGTCTCTTCTGCACC
	EGFR CE8 CRISPR KRAB 4 Fw	CACCGGAAATTCTTCCCCTACGAG
	EGFR CE8 CRISPR KRAB 4 Rv	AAACCTCGTAGGGGAAGAATTTCC

Supplementary Table S4

Purpose	Primer Name	Primer Sequence
Genotyping	EGFR CE5B Genotyping1 Fw	ACACAAAACCCTCAGGTGGT
	EGFR CE5B Genotyping1 Rv	AGTGGGGAAAATGGACTCTGA
	EGFR CE5B Genotyping2 Fw	TTCGCACATCACAGAGGCAT
	EGFR CE5B Genotyping2 Rv	GTCTCTGTGGATGCATGGTT
	EGFR CE6B Genotyping1 Fw	ACACCAACAGAAGACAGCCA
	EGFR CE6B Genotyping1 Rv	GAACGTGCTTTTGTCCGTGA
	EGFR CE6B Genotyping2 Fw	TATCGTCTTGCTTGCTCCCC
	EGFR CE6B Genotyping2 Rv	ACACCCTTTGGCCTTCTATTCA
	EGFR CE8 Genotyping1 Fw	CTCTCCTGAGGGTGGTCTGA
	EGFR CE8 Genotyping1 Rv	GTCTGACTCCCCACTGCTTC
	EGFR CE8 Genotyping2 Fw	TGCCAGATGTGAACAAGGGG
	EGFR CE8 Genotyping2 Rv	GGGCAGTACTACAAAGCGGA
	EGFR Prom Genotyping1 Fw	TACAGCTGGCAAAGGGATGG
	EGFR Prom Genotyping1 Rv	CTGTGGAGGGTGGTCCTAGA
	EGFR Prom Genotyping2 Fw	TCTAAAAGCACCTCCACGGC
	EGFR Prom Genotyping2 Rv	TGTCCAGGTCGAGCCAAATC

Supplementary Table S5

Purpose	Primer Name	Primer Sequence
RT-qPCR	EGFR Fw hu qPCR	TATTGATCGGGAGAGCCGGA
	EGFR Rv hu qPCR	TCGTGCCTTGGCAAACTTTC
	HPRT Hu qPCR Fw	CATTATGCTGAGGATTTGGAAAGG
	HPRT Hu qPCR Rv	CTTGAGCACACAGAGGGCTACA
	CS Fw	CACAGGGTATCAGCCGAACCAA
	CS Rv	CCAATACCGCTGCCTTCTCTGT
	IDH1 Fw	CTATGATGGTGACGTGCAGTCG
	IDH1 Rv	CCTCTGCTTCTACTGTCTTGCC
	IDH2 Fw	AGATGGCAGTGGTGTCAAGGAG
	IDH2 Rv	CTGGATGGCATACTGGAAGCAG
	IDH3A Fw	TCGGTGTGACACCAAGTGGCAA
	IDH3A Rv	TTCGCCATGTCCTTGCCTGCAA
	OGDH Fw	GAGGCTGTCATGTACGTGTGCA
	OGDH Rv	TACATGAGCGGCTGCGTGAACA
	FH Fw	CCGCTGAAGTAAACCAGGATTATG
	FH Rv	ATCCAGTCTGCCATACCACGAG

MDH1 Fw	CGGTGTCCTAATGGAACTGCAAG
MDH1 Rv	CATCCAGGTCTTTGAAGGCAACG
MDH2 Fw	CTGGACATCGTCAGAGCCAACA
MDH2 Rv	GGATGATGGTCTTCCCAGCATG
HK1 Fw	CTGCTGGTGAAAATCCGTAGTGG
HK1 Rv	GTCCAAGAAGTCAGAGATGCAGG
HK2 Fw	GAGTTTGACCTGGATGTGGTTGC
HK2 Rv	CCTCCATGTAGCAGGCATTGCT
PFKL Fw	AAGAAGTAGGCTGGCACGACGT
PFKL Rv	GCGGATGTTCTCCACAATGGAC
PFKM Fw	GCTTCTAGCTCATGTCAGACCC
PFKM Rv	CCAATCCTCACAGTGGAGCGAA
PFKP Fw	AGGCAGTCATCGCCTTGCTAGA
PFKP Rv	ATCGCCTTCTGCACATCCTGAG
PKM2 Fw	ATGGCTGACACATTCCTGGAGC
PKM2 Rv	CCTTCAACGTCTCCACTGATCG
NDUFA2 Fw	TACGTGGAGCTGAAGAAGGCGA
NDUFA2 Rv	ACGTTCTCCAGGGCTCTGGTTA
SDHA Fw	GAGATGTGGTGTCTCGGTCCAT
SDHA Rv	GCTGTCTCTGAAATGCCAGGCA
CYC1 Fw	CCAGATAGCCAAGGATGTGTGC
CYC1 Rv	GACTGACCACTTGTGCCGCTTT
PDHA1 Fw	GGATGGTGAACAGCAATCTTGCC
PDHA1 Rv	TCGCTGGAGTAGATGTGGTAGC
LDHA Fw	GGATCTCCAACATGGCAGCCTT
LDHA Rv	AGACGGCTTTCTCCCTCTTGCT
LDHB Fw	GGACAAGTTGGTATGGCGTGTG
LDHB Rv	AAGCTCCCATGCTGCAGATCCA
